# Effects of the Consumption of Prickly Pear Cacti (*Opuntia spp.*) and its Products on Blood Glucose Levels and Insulin: A Systematic Review

**DOI:** 10.3390/medicina55050138

**Published:** 2019-05-15

**Authors:** Caroline A. Gouws, Ekavi N. Georgousopoulou, Duane D. Mellor, Andrew McKune, Nenad Naumovski

**Affiliations:** 1 Faculty of Health, University of Canberra, Bruce, ACT 2617, Australia; caroline.gouws@canberra.edu.au (C.A.G.); andrew.mckune@canberra.edu.au (A.M.); 2 Collaborative Research in Bioactives and Biomarkers Group, Bruce, ACT 2617, Australia; ekavi.georgousopoulou@anu.edu.au (E.N.G.); duanemellor@btinternet.com (D.D.M.); 3 Medical School, Australian National University, Canberra, ACT 2605, Australia; 4 School of Life Sciences, Coventry University, Coventry CV1 2DS, UK; 5 Discipline of Biokinetics, Exercise and Leisure Sciences, School of Health Sciences, University of KwaZulu-Natal, Durban, KwaZulu-Natal 4000, South Africa

**Keywords:** *Opuntia*, prickly pear, cactus pear, cladode, glucose, insulin, human, systematic review

## Abstract

*Background and Objectives*: There is confusion as to which component of the *Opuntia spp.* cacti has demonstrated anti-hyperglycemic effects or anti-diabetic properties. It is important to clarify these health benefits due to the increasing need for prevention and treatment of chronic diseases. The aim of this review is to identify the effects of *Opuntia spp.* cacti consumption on biomedical measures; glucose and insulin with consideration of its’ components; fruit, leaf and combined or unidentified *Opuntia spp.* products. *Materials and Methods*: Prior to commencing the searches, this systematic review was registered with PROSPERO (CRD42018108765). Following the PRISMA 2009 guidelines, six electronic databases (Food Science and Technology Abstracts (EBSCO), Medline, Scopus, CINAHL, Web of Science and Cochrane) were searched for articles investigating the effect of *Opuntia spp.* consumption on glucose and insulin in humans. *Results*: Initially, 335 articles were sourced and filtered by exclusion criteria (human interventions, control trials and articles published in English) resulting in 20 relevant articles. The included studies were characterized by such plant components as fruit (*n* = 4), cladode (*n* = 12), and other *Opuntia spp.* products (*n* = 4), further separated by clinical populations (‘healthy’, hyperlipidemic, hypercholesterolemic, Type 2 Diabetes Mellitus). The findings of this review indicate variations in effects between cacti components and products. Cladode and select *Opuntia spp.* products predominately demonstrated significant reductions in serum glucose and insulin, indicating potential as a functional food candidate. Prickly Pear fruit was predominately reported to have no significant effects on glucose or insulin. The quality of evidence appeared to vary based on the type of *Opuntia spp.* product used. Studies that used specifically the fruit or cladode had high risk of bias, whereas studies which used combined *Opuntia spp.* products had a lower risk of bias. Numerous mechanisms of action were proposed where positive findings were reported, with emphasis on dualistic glucose-dependent and independent actions, however, mechanisms require further elucidation. *Conclusion*: Currently, there is a lack of evidence to support the recommendation of using *Opuntia spp.* fruit products as an alternative or complementary therapy in the reduction of risk or management of Type 2 Diabetes Mellitus. The Cladode does however show promise in potential glucose-lowering effects which warrant further investigation.

## 1. Introduction

The prevalence of Type 2 Diabetes Mellitus (T2DM) and the corresponding demand for its management is proving to be a global economic burden [1]. Various strategies, including dietary management, exercise-based therapies, novel drug development, personalized medicine, pharmacotherapy, pathogenesis, traditional medicines, and combined anti-diabetic or ‘anti-hyperglycemic’ therapies are prioritized as various methods for treatment and prevention of T2DM globally [1].

Herbal treatments and traditional plant-based medicines have become increasingly popular, as convenient and low-cost options with potentially fewer side effects [1]. Herbal or plant-based anti-hyperglycemic treatments are reportedly targeted to biochemical pathways, including antioxidant/free radical pathways based on hyperglycemia being associated with mitochondria overproduction of free radicals [1,2]. Additionally, there is increasing interest in management strategies for T2DM and its prevention based on alternative low-cost nutrition strategies, including plant-based functional-foods [2]. However, currently, consensus has only been reached regarding the anti-hyperglycemic effect relating to the high-fiber content of many of the proposed anti-hyperglycemic functional foods. This is primarily based on the fiber content slowing the rate of digestion and the resultant altered rate of glucose absorption [2].

Worldwide, there is a focus on sustainable and responsible preventative health interventions, with the emphasis on socio-political, economic and environmental factors [3]. The social push to include environmental factors has led to the consideration of the effectiveness of low-resource intensive plant-based treatments for the maintenance and prevention of T2DM. This includes the potential therapeutics derived from cacti [3,4]. Specifically, *Opuntia spp.* cacti, with a strong history of use as a treatment of hyperglycemia in traditional medicine [4], will be the focus of this systematic review. The *Opuntia spp.* cacti is utilized as a source of food, hedge plant, or as an ornamental plant, particularly in arid and drought-affected regions, becoming more relevant with environmental cases of desertification [5,6,7,8]. The cacti as a crop is utilized, particularly on non-productive agricultural lands, for its resources, that include the fruit, the Prickly Pear (PP) and leaf, the cladode [5,6,7,8]. The *Opuntia spp.* cacti is reported to have ‘anti-hyperglycemic’ and ‘anti-diabetic’ properties in humans and it is prescribed in many traditional and complementary therapies around the world [9,10,11,12]. However, confusion exists within the literature as to which principal cacti component is potentially responsible for such effects. Clarification of this issue could enhance the use of PP for treatment, maintenance, and prevention of chronic diseases and affect progress in the development of new potential anti-hyperglycemic agents.

To be able to further consider *Opuntia spp.* components for new anti-hyperglycemic agents, there is a need to identify which component(s), if any, are the true source of any potential anti-hyperglycemic effects of *Opuntia spp*. There are obvious compositional variations between the leaf and fruit which require investigation to define any possible resultant health associations [4]. The fruit of the cacti is also referred to as the ‘cactus fruit’ or ‘Indian fig’ and also as the PP [6,7] is predominantly known for relatively high levels of macro- and micronutrients including fiber, mineral composition, numerous amino acids; and significant lipid and phytosterol content [13,14,15,16,17]. In addition, there are a number of different bioactive compounds found in PP such as polyphenols [18,19], flavonols [19,20,21] and betalains [18,22,23], as well as considerably strong antioxidant characteristics [17,18,20,21,24,25,26,27,28,29,30,31]. The PP fruit is commonly consumed fresh, although it can be formulated into several different including dried fruits, tea’s, jams, drinks, tinctures and dietary supplements [6,32,33].

The *Opuntia spp.* cacti leaves, often termed the ‘cladode’ are commonly eaten as a broiled or grilled food product. The cladodes’ compositional content varies substantially from the fruit, although it does share common features to include high fiber, mineral, and selection of different bioactives [34]. Similar to PP, the cladode has been extensively investigated for bioactive compounds to include extensive phenolic analysis and antioxidant characterization of various extracts [34]. More recently, the cladode was used in various forms in the food and supplements market such as various pickled products, fiber powders, or capsule-based supplements [35,36].

Therefore, based on the current findings, the primary aim of this systematic literature review is to examine the evidence drawn from well-controlled human clinical trials on the effect of consumption on *Opuntia spp.* cacti on blood glucose (GLU) and insulin levels (INS) in humans. The outcomes of this systematic review will potentially assist clinicians with decisions regarding the use of *Opuntia spp*. product (fruit, leaf or combined) consumption, for improvement of T2DM associated clinical measures such as hyperglycemia or insulin regulation.

## 2. Materials and Methods

**Figure 1:**
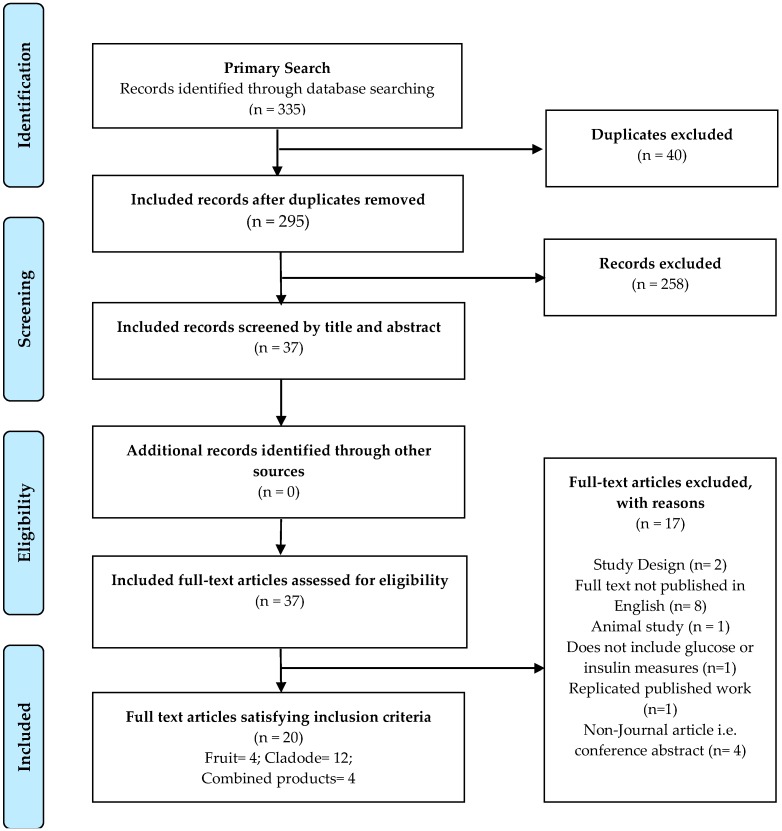
PRISMA Flow chart of inclusion and exclusions of searched database outputs.

This systematic literature review was conducted following the PRISMA 2009 guidelines [37], and searches were performed in six electronic databases: Food Science and Technology Abstracts (EBSCO), Medline, Scopus, CINAHL, Web of Science and Cochrane databases. The generated results were screened for relevance by title, abstracts and full text (Figure 1), with additional hand searches of included articles reference lists. Results were limited to human quantitative intervention trials based on the consumption of *Opuntia spp.*, investigating the effects on blood GLU or INS resistance parameters, published in English, in peer-reviewed journals since the journal inception. No limitations were made regarding age, gender, race or ethnicity. This systematic literature review is also registered with the PROSPERO registry of the systematic literature reviews held at the University of York (CRD42018108765).

### 2.1. Search Terminology and Selection Criteria

The following search terms were used, to produce the database outputs: “(‘Opuntia’ OR “Prickly Pear” OR “Cactus fruit” OR “Tuna fruit” OR “Indian fig” OR ‘Nopal’ OR ‘Cladode’ OR “Cactus Leaf” OR ‘Stems’) AND (‘Glucose’ OR ‘Blood glucose’ OR ‘Glucose tolerance’ OR ‘Impaired glucose’ OR ‘Oral glucose’ OR ‘Glucose metabolism’ OR ‘glycemia’ OR ‘Glycaemia’ OR ‘Hyperglycemia’ OR ‘Fasting blood glucose’ OR ‘Glycemic Index’ OR ‘HOMA’ OR ‘QUICKI’ OR ‘GI’ OR ‘Insulin’ OR ‘Insulin resistance’ OR ‘Plasma insulin’ OR ‘Insulin secretion’). Eligibility for inclusion was determined via satisfaction of criteria; human, in vivo, quantitative intervention trials with control groups or placebo, consumption of *Opuntia spp.* and published since journal inception until September 2018 (5th). The reference lists of included articles were searched for similar phrases and screened for relevance.

### 2.2. Data Extraction and Outcomes of Interest

Data extraction was conducted independently by two reviewers (C.G., and N.N.) who assessed the eligibility of imported EndNote (v8, Thomas Reuters, Eagan, MN, USA) citations from the database outputs, after removing duplicates. Excluded articles within the screening of titles, abstracts, and full-text articles were excluded on consensus, where disagreement was resolved via discussion. In total, 37 full-text articles were selected for review; and only 20 were included in this systematic review. Prompting reference list searches of included articles, no further articles were identified as suitable for inclusion in this review. Primary outcomes of interest for this systematic review included measures of blood or serum GLU and INS, including biochemical assays, or well-known tools, Homeostatic Model Assessment (HOMA) and ‘Quantitative Insulin Sensitivity Check Index’ (QUICKI).

### 2.3. Data Analysis

Overall studies were evaluated on; risk of bias; study characteristics to include sample size; and participant characteristics including age and gender, study design and intervention. More specifically tools of analysis and reported outcomes were also analyzed to make meaningful conclusions. Due to the heterogenicity of different food products and non-quantifiable composition of some macro- and micronutrients in the provided treatments (food and/or supplement) a meta-analysis was not deemed suitable.

### 2.4. Risk of Bias

The risk of bias for each included study was evaluated using the ‘Cochrane Risk of Bias Tool’ [38]. The forms of bias considered included; selection, performance, detection, attribution, reporting and ‘other’ biases, which were scored either ‘low’, ‘high’ or ‘unclear’.

## 3. Results

### 3.1. Search in Literature

Of the 335 results, 20 articles met the inclusion criteria (Figure 1). After removal of duplicates, the included articles were substantially reduced by title and abstract relevance screening (258 removed). Searches of the included article reference lists which did not yield any additional studies were unsuccessful in recruiting additional literature for inclusion. Remaining articles were progressed to full-text screening, where results were filtered to include 20 manuscripts investigating consumption associated effects of various components of the *Opuntia spp*. on blood GLU and INS resistance measures. Of the included studies; four studies [39,40,41,42] investigated the *Opuntia spp*. fruit (Table 1), twelve studies [43,44,45,46,47,48,49,50,51,52,53,54,55] investigated the *Opuntia spp*. cladode (Table 2), and four studies [36,51,56,57] investigated unspecified or combinations of both the *Opuntia spp*. fruit and cladode (Table 3).

The included studies were scored for risk of bias (Table 4) by two researchers (C.G. and N.N.) following the Cochrane Risk of Bias Tool [38]. Investigations into single plant products (fruit; cladode) tended to have a high selection (3; 3) and performance bias (3; 9), with the exception of Wiese et al. (2004) [41] and Bacardi-Gascon et al. (2007) [55]. Across all included studies, detection bias was rated as high, or unclear, if not detailed within the methodology of the article. Reporting bias was consistently low for all included studies.

### 3.2. Results of Included Studies

#### 3.2.1. Results of *Opuntia spp*. Fruit Included Studies

The studies included in the category of the PP fruit (Table 1), utilized various forms such fruit pulp [40], the juice from the fruit pulp [39], fruit peel [42] and fruit extracts [41]. Nearly all of these studies were conducted in males, except for one study conducted in females *(n* = 10 female; T2DM) [42] and one in males and females (*n* = 55; 18 female; 37 male) [41]. Typically, studies in this category were matched with placebo treatments (capsule) [41] or control treatments (GLU control solution, dietary patterns) [39,40,42] and duration of treatment ranged from single consumption [41,42], short term (2–5 weeks) [39,42] and long term (16 weeks) [40].

Supplementation with the purple PP fruit pulp juice over the period of two weeks [39] resulted in significant differences in fasting GLU levels in ‘healthy’ males undergoing an exercise intervention (*p* = 0.01). Wolfram et al. (2002) [40] using a PP fruit pulp (250 g/day; 8 weeks) also reported significant decreases in fasting GLU (*p* < 0.005) of participants with hypercholesterolemia and hyperlipidemia; and INS (*p* < 0.005) in participants with hypercholesterolemia but not hyperlipidemia (*p* > 0.05). Pimienta et al. (2008) [42] reported no significant effects of PP fruit after a single consumption of PP fruit peel, in ‘healthy’ subjects (*n* = 14; *p* > 0.05). Similarly, there were no significant lowering effects inT2DM participants (*n* = 10) after 5 weeks of treatment for both blood GLU and INS (Both *p* > 0.05), using Oral GLU Tolerance Tests (OGTT) and ‘pre-’ and ‘post-’ intervention serum samples. In addition, Wiese et al. [41] examined the effect of commercially available PP fruit extract capsules on reducing symptom severity in ‘healthy’ participants with induced alcohol toxicity and reported no significant differences between treatment and placebo on serum GLU (*p* > 0.05).

#### 3.2.2. Results of *Opuntia spp*. Cladode Included Studies

Studies on the cladode of *Opuntia spp.* included numerous preparations of these products as a food delivery method such as; cooked (broiled, grilled) [43,44,47,54], powders [45,49,50], capsules [52,53] and new products such as tortillas including the cladode [55]. The cladode was studied in; ‘healthy’ [43,44,45,46,48,49,50,51,52], obese [46,52], T2DM [43,44,45,46,47,48,50,54,55] participants as control [45,46,47,48,49,50,51,52] and cross-over trials [50,53,54,55]. In most of the studies, intervention was matched with a placebo [52] or control interventions [43,45,46,47,48,49,50,53,54,55]. The interventions were predominantly based on single consumption [43,44,45,47,48,50,54], although two studies included short term (2–3 weeks) [53,55] and two studies included long terms (6–10 weeks) [46,52] treatments.

Interestingly, majority of the studies reported in this systematic literature review using the cladode as a food delivery product were from Frati and colleagues, where seven articles were included as a part of a separate trial. Firstly, a study by Frati et al. (1983a) demonstrated significant effects in reduction of GLU (*p* < 0.01–0.05) and INS (*p* < 0.01–0.02) post-prandial (0–3 h) with ingestion of 100g of broiled cladode in healthy males [45]. When consumed for a longer period (10 days), a study by Frati et al. (1983b) [46] indicated that consumption of the previously established quantity of cladode (100g) had significant reductions in fasted serum GLU (mean: 3.5 mmol/L) for T2DM participants (*n* = 7; *p* < 0.01). Additionally, significant reductions were also reported in ‘non-diabetic’ participants (mean: 0.021 mmol/L *p* < 0.05). In some cases, the participants of certain comparisons were not defined to the reported study groups but rather generalized as ‘healthy’ (*n* = 8) and ‘obese’ (*n* = 14; combined study groups) [46]. A study that followed [48] built on the single consumption investigations, where Frati et al. (1987) considered the effects of treatment and OGTT protocol interactions (cladode; cladode before dextrose bolus; cladode mixed with dextrose) to find significantly lower serum GLU (*p* < 0.025; vs. water) at 60 and 180 min in healthy adults. No significant (*p* > 0.05) effects were reported for serum INS between the treatments [48]. Comparisons between single consumption of *Opuntia spp.* cladode and zucchini squash (500g) in T2DM participants (*n* = 48) were drawn on both GLU and INS parameters [47]. Consumption of the broiled cladode resulted in significant reductions in serum GLU (1 h 8.5 ± 2.4%; 2 h 10.7 ± 1.4%; 3 h 17.6 ± 2.2% µU/mL; *p* < 0.01–0.025) and INS (*p* < 0.001; 1 h 22.5 ± 5.2%; 2 h 38.7 ± 6.3%; 3 50.2 ± 8.0%) [47].

A study by Frati et al. (1990) [53] continued to examine the effects of various cladode processing techniques [48] (broiled, raw blended, broiled and blended, blended and broiled; 500 g) on fasted serums GLU in healthy participants. This study it was reported significant reductions in 120 and 190 min (*p* < 0.001), although no significant (*p* > 0.05) effects were observed between processing techniques [58]. Investigations into the effects of cladode consumption with established volume and preparation on GLU and INS continued into T2DM participants, whereby the timing of sequential consumption was considered [44]. In this study, there was a significant reduction (*p* < 0.01) is fasting serum GLU in T2DM participants (2 h, 36.8 mg/dl; 4 h, 92 mg/dl; 6 h, 49 mg/dl) but not in healthy participants (*p* > 0.05) [44]. A study examining the effect of grilled cladode (500 g) in matched T2DM (*n* = 14) and healthy (*n* = 14) participants reported significant reductions in GLU (1 h: 1.19 ± 0.21 mM, *p* < 0.005; 2 h: 1.57 ± 0.28 mM, *p* < 0.005; 3 h: 2.26 ± 0.25 mM, *p* < 0.001) and INS (*p* <0.01; 1 h: 12.3 ± 15.9; 2 h: 3 h: 36.9 ± 10.8 pM; 56.4 ± 10.8 pM) in T2DM but not in a healthy group [43]. These studies indicate that the consumption of cladode prepared in various ways (cooking procedures) significantly reduced blood GLU and INS levels in T2DM participants. Despite the significant reductions of GLU and INS with consumption of *Opuntia spp.* Cladode in T2DM participants, there is limited evidence to support the beneficial effects of the vegetable consumption in ‘healthy’ participants.

The remaining included articles considering cladode products in different forms such as an extract in a capsule [52,53] a meal replacement [49] and in combination with food [49,50,55]. Castaneda-Andrad et al. (1997) [53] reported that single dose cladode consumption, using a capsule (250 mg) significantly decreased serum GLU (319–220 mg/dL) in individuals living with T2DM compared with placebo (*p* < 0.01). In addition, 6 weeks of supplementation of cladode extracts, provided as a capsule (*‘NeOpuntia’*; 3 × 1.6 g), had no effect on glucose associated risk factors of Metabolic Syndrome in a female cohort [52].

Cladode was also studied in combination with other foods, and it was provided in addition to foods incorporated or provided in a blinded form. A study by Lopez-Romero et al. (2014) [50] conducted two trials within the same paper. In the first experiment, these authors investigated the glycemic responses to consumption of cladode powder (50 g) in healthy participants. The powder significantly lowered serum GLU (*p* < 0.001) and produced a glycemic index score of 32.5 ± 4.0, and insulinemic index of 36.1 ± 6.1, upon ingestion. In the second experiment, the effect of cladode consumption in combination with various breakfasts (high carbohydrate; high soy protein) on serum GLU and INS in T2DM (*n* = 14) and healthy participants (*n* = 7) was investigated [50]. The results showed that T2DM participants nopal consumption in combination with a high carbohydrate breakfast incurred significant decreases in serum GLU (30 min, 45 min and 1 h; *p* < 0.01), GLU by Area Under the Curve (AUC reduction 287 ± 30 mmol/L; *p* < 0.001), with no significant differences reported in serum INS [50]. Significant differences were also observed between high carbohydrate and high soy protein breakfasts in GLU levels (*p* < 0.001).

Lopez-Romero et al. (2014) reported no significant differences (*p* > 0.05) in cladode powder ingested with a high-soy protein breakfast for parameters of GLU or INS in participants with T2DM, but there was a significant difference for INS AUC in healthy participants (decrease; *p* < 0.01). A study by Bacardi-Gascon et al. (2007) [55] investigated the cladode consumption by incorporating it into three meals; chilaquiles, burritos, and quesadillas with or without the cladode. Significant decreases in serum GLU (AUC) were apparent in all three meals (chilaquiles *p* = 0.018; burritos *p* = 0.025; quesadillas *p* = 0.019). A separate study by Guevare-Cruz et al. (2012) investigated the effects of a dietary pattern containing cladode among other ingredients to include chia seeds, oats, and soy protein against a less nutrient and bioactive dense placebo dietary pattern over a 2-month period [49]. The mentioned dietary pattern was found to have a significant difference on AUC for INS (*p* < 0.05) and diet-time interactions for INS as well (*p* < 0.001). The GLU lowering effects of *Opuntia spp.* of the cladode are supported by literature; however, more evidence is recruited to make conclusions regarding INS.

#### 3.2.3. Results of Combined or Unspecified *Opuntia spp*. Included Studies

Combined *Opuntia spp*. plant products *(*Table 3*)* were studied in both males and females, using the specifically-formulated tortillas and bars [51] or commercially available ‘OpundDia^©^’ capsule dietary supplement, reported to contain 75% Cladode and 25% PP fruit extract. The *Opuntia spp.* products were investigated with control [36,51] and cross-over [56,57] designed trials in healthy [51,56,57] and obese [36] participants. Investigations into combined fruit and cladode products were consistent, where controls were established as placebo capsules [36,56,57] or formulated products for investigation into single consumption models.

Investigations considering combined *Opuntia spp.* products considered the efficacy of the established products, with the exception of Guevera-Arauza et al. (2011) [51], who developed several products. Acute and chronic effects of ‘OpunDia^©^*’* in obese participants were investigated by Godard et al. (2010) [36]. Acute trial (*n* = 29; 400 mg) indicated significant differences in serum GLU for time points 1, 2, and 3 h (*p* < 0.05) between treatment and control (placebo capsules). Guevera-Arauza et al. (2011) [51] concluded that the chronic supplementation of ‘OpunDia*^©^*’ over 16 weeks induced significant reductions in GLU both pre-and post-treatment (*p* < 0.05) and placebo (*p* < 0.05); and had no significant effects on INS [36].

A study by Van Proeyen et al. (2012) [57] investigated dose of ‘OpunDia^©^’ treatments (500 mg, 1000 mg, and 1500 mg) in combination with exercise interventions. Significant reductions in serum GLU were reported in pre-exercise (30 and 60 min; *p* < 0.10–0.02) and post-exercise (60 min; *p* < 0.04); and significantly increases INS during pre-exercise at 30 min (*p* < 0.03), but not post-exercise [57]. A study by Deldicque et al. (2013) [56] continued in active participants, investigating the interaction between ‘OpunDia^©^’ in combination with amino acid, leucine, on INS stimulation in ‘healthy’ males following (endurance) exercise [56]. In this study, there was a significant increase in INS with ‘OpunDia^©^’ (*p* < 0.05) and in combination with Leucine (*p* < 0.05); and significant decreases in GLU with ‘OpunDia^©^’ (*p* < 0.05) but no other synergistic combinations.

A study by Guevera-Arauza et al. (2011) [51] differed from other included combined *Opuntia spp.* in that the *Opuntia spp.* products were developed by investigators for the purpose of the study. The aim of this study was to determine the effect of the *Opuntia spp.* PP fruit and cladode in placebo and fortified food products including various bars and tortillas were tested on serum GLU. The results demonstrated a significant lowering effect (*p* < 0.05; 4.43 mmol/L) on serum GLU in healthy participants upon product consumption.

## 4. Discussion

The *Opuntia spp.* cacti and its components have been reported to have ‘anti-diabetic’ or ‘anti-hyperglycemic’ properties [56]. However, confusion exists within the literature between which *Opuntia spp.* components are responsible for the reported health effects [34,56]. The *Opuntia spp.* cladode demonstrated significant positive health effects, indicating its potential as a functional food for potential short-term treatment of hyperglycemia in multiple sample participants.

Proposed mechanisms underlying how plant-based products may induce anti-hyperglycemic effects include individual or combined effects via the modulation of gastrointestinal absorption or regulation of INS secretion and sensitivity [56]. The anti-hyperglycemic activities provided by plant-based products are diverse and are a result of a multiple contributing factors, including the presence of INS-like substances or ‘factors’ within the plant material [59], upregulation of INS via β-cells [60], increased insulin binding activity of receptors [60], increase GLU metabolism [61], high fiber content reducing the rate of absorption [62], or potential regeneration of pancreatic cells [63]. Various aspects of potential mechanisms are proposed following positive effects of specific *Opuntia spp.* products reported within this review. The findings of this review provide rationale for the use of *Opuntia spp.* products in applications such as incorporation into functional foods or nutraceuticals, given the additional substantial bioactive contents [18,19,20,21,22,23,24,25,26,27,28,29,30,31].

### 4.1. Opuntia Ficus Indica Fruit

Overall, the results of this systematic review suggest no significant effects on blood GLU [39,41,42] and INS [42], upon investigations into *Opuntia ssp.* PP fruit pulp [40], pulp juice [39], fruit peel [42], and fruit extracts [41]. The conclusions made regarding the results of this systematic review do consider the disagreements of significant differences in the reduction of GLU in hypercholesterolemic (*n* = 12) and hyperlipidemic (*n* = 12) sample participants; and INS in a hypercholesterolemic sample population (*n* = 12) [40]. Although serum GLU was investigated in every included study regarding the PP fruit, it is important to recognize that INS was only studied in two of the four included fruit-based investigations [40,42]. The conclusions drawn with respect to *Opuntia spp.* fruit are limited by high risk of bias with relation to selection, performance, and detection biases, but did show strengths in attribution and reporting bias.

Interestingly, the widely accepted associations of fiber as an anti-hyperglycemic effect [62] were observed within the fruit-based studies included in this review. Anti-hyperglycemic effects are often reported in trial foods with a higher fiber content [62], such as that expected of fruit flesh, unlike a majority of the investigations within this review. The included fruit-based studies investigating fruit products with substantially less fiber to include; juice [39], peel [42] and extracts [41]. Future research should investigate the effects of PP fruit consumption regarding the INS-GLU relationship, with consideration of fiber. The results of this review do not encourage the prescription of PP fruit as an alternative medicine in treatment of hyperglycemia or associated conditions, until further evidence is developed.

### 4.2. Opuntia Ficus Indica Cladode Leaf

*Opuntia spp.* cladode consumption was associated with significantly lower serum GLU in healthy, obese [46,52], and individuals with T2DM [43,44,46,47,50,53,54,55]. Of the 12 included cladode-based investigations, 6 studies investigated INS responses [43,45,47,48,49,50]. Conclusions regarding INS were not definitive, due to conflicting evidence [43]. The majority of the sourced results indicated significant changes in serum INS on cladode consumption in healthy participants [45] and individuals with T2DM [43,47,50]. Conflicting evidence with respect to INS was seen in active and placebo diets including the cladode, as a whole product [50] or as a powder (*n* = 67) [49]; and studies investigating interactions between OGTT timing and cladode consumption, also indicated no significant effects [48].

Consistent with previous hypotheses regarding GLU lowering mechanisms, the cladode leaf is reported to contain substantial amounts of fiber [34,62]. Currently, due to the cladode’s fiber content, it is utilized for the development of many food products and supplements, such as flours and fiber supplements [34]. Future research should consider investigation into other potential mechanisms of action, and inter-relationships should be considered regarding the anti-hyperglycemic effects observed via consumption. In conclusion, the consumption of the cladode has noticeable effects of hyperglycemia; however, it should be recommended with caution and consideration of commonly-associated effects of food with high fiber content.

### 4.3. Opuntia Ficus Indica as Combined Fruit and Cladode Leaf

Of the included articles within this review, four were identified as combined *Opuntia spp.* products; ‘OpunDia^©^*’* capsule-based investigations [36,56,57] and cladode-based food products [49]. Both *Opuntia spp.* product investigations reported significant reduction in serum GLU. To our knowledge, the literature surrounding the effects of *Opuntia spp.* products on INS, were only studied using the ‘OpunDia^©^’ capsule; however, they reported conflicting results [36,56,57].

The beneficial effects of the combined supplements (cladode and fruit) may be due to the greater cladode to fruit proportions within the ‘OpunDia^©^’ capsules. Noticeably, the studies examining the effects of the combined *Opuntia spp.* products do not control for the anti-hyperglycemic effects of fiber [62]. A study by Deldicque et al. (2013) [56] based investigation into ‘OpunDia^©^’ on the proposed mechanism of direct upregulation of INS by the *Opuntia ficus indica spp*, rather than indirect production of INS, in response to increased serum GLU. Multiple studies propose the direct insulinogenic actions of the *Opuntia spp.* products, described as acute and rapidly acting [56], a result of upregulation of pancreatic β-cell INS secretion [56,57]. Although, a study by Van Proeyen et al. (2012) [57] proposed a dualist mechanism stemming from both GLU-induced and GLU-independent INS production of the combined *Opuntia spp.* product consumption. However, this finding was in a population undergoing an exercise intervention, in which they were characteristically more susceptible, or had improved INS sensitivity [57]. The use and effectiveness of combined *Opuntia spp.* products are largely specific to the product, where further evidence is required on mechanisms of action before making conclusions regarding the effectiveness or advised supplementation.

## 5. Conclusions

The findings of this systematic literature review indicate that the cladode leaf and selection of combined *Opuntia spp.* products (typically containing a mix of cladode leaf and fruit) may have significant effects of serum GLU reduction. However, effects on INS were less consistent. The PP fruit was found to not have significant ‘anti-hyperglycemic’ effects, though the definitive evidence was conflicting. This suggests that there might be variability in the potential anti-hyperglycemic effects of different plant parts of *Opuntia spp*., which warrants further investigation. Study characteristics including design, exposure, quantity, and participants, as well as the treatment product and compositions, varied extensively, limiting the conclusions within this review. Future research should investigate the serum glucose-insulin relationships within consumption-based investigation and the inter-relationships within the proposed mechanisms of action, ideally following standardized and reproducible methodologies.

## Figures and Tables

**Table 1:** The Summary of the effect of *Opuntia spp*. fruit consumption on blood glucose levels and insulin in human trials [39,40,41,42].

Author (Year)	Participant, Sample Size	Aim	Intervention	Relevant Outcome	Results (C vs. T))
Khouloud et al. (2016) [39]	Healthy M (*n* = 22) T (*n* = 11) Age: 20.91 ± 1.22 years C (*n =* 11) Age: 21.00 ± 0.84 years	Effect of flavonoid standardized PP fruit (*Opuntia ficus indica*; purple; peeled*)* juice consumption on cardiovascular, oxidative stress and biochemical parameters during exercise	Duration: 2 w T: 3 × day 30 mL/day PP juice; C*:* No antioxidants: 150 mL/day RCT	Fasted GLU	Before vs. After Exercise: ↑ GLU (*p* = 0.01) for both Treatment and Control groups No difference between groups were reported
Wolfram et al. (2002) [40]	M (*n* = 24) Age: 37–55 y (45 ± 4.7 years) Group 1: (*n* = 12) ‘Hypercholesterolemic’ Group 2: (*n* = 12) ‘Hyperlipidemic’	The effect of PP (*Opuntia Robusta)* pulp pectin on GLU and lipid metabolism	Duration: 16 w Pre-running phase: Diet: 7506 kJ (625 kJ 50% fibers and 50% CHO; 8 w) Phase 2: Replaced diet fiber and CHO with fresh 250g/day of PP pulp (8 weeks) Parallel CT	Fasted (14 hrs.): GLU INS	↓ GLU between phase 1 and 2 (*p* < 0.005) for both groups 1 and 2 ↓ INS between phase 1 and 2 for group 1 (*p* < 0.005) INS group 2 = NS (*p* > 0.05)
Pimienta et al. (2008) [42]	Phase 1: ‘Healthy’ M (*n* = 14) Age: 22.4 ± 3.2 years Phase 2: ‘T2DM participants’ F (*n =* 10) Age: 42.4 ± 3.3 years	The effects of yellow PP fruit peel on health males (single consumption) and diabetic females(Chronic; 5 weeks)	Phase 1: Single consumption *Treatment:* 250 g fruit peel and GLU solution *Control:* 75 g GLU (solution) Control trial Phase 2: Duration: 5 w Fasted; 3 × 50 g PP peel/week *Control:* Baseline measurements Control Trial	OGTT (12 h fasted) Time −20, 0, 20, 30, 60, 80, 100, 120, 140, 160, 190, 200 min Phase 1 and 2: GLU INS	Phase 1: ↓ GLU: treatment vs. control (40 min) (*p* < 0.05), all other time points NS (*p* > 0.05) ↑ INS treatment NS (*p* > 0.05) Phase 2: INS ‘healthy’ and diabetics NS (*p* > 0.05) GLU: diabetics NS (*p* > 0.05)
Wiese et al. (2004) [41]	‘Healthy M&F with history of at least one hangover (*n* = 55) M (*n* = 37) F (*n* = 18) Age: 21–25 years	The effect of *Opuntia ficus indica* PP fruit extract in reducing symptoms of severity from alcohol hangover	Length: Single consumption *Induced hangover:* Consumption of 1.75 g of alcohol/kg (Low: vodka, gin, rum; high: bourbon, scotch, tequila) with standard meal (cheeseburger, French fries and soda) Treatment: 2× capsule of *Opuntia ficus indica* PP fruit extract Placebo: 2× capsule 2 w washout Randomized Control Cross-Over Trial	GLU	Baseline VS. placebo: NS (*p* > 0.05) Placebo VS. treatment: NS (*p* > 0.05)

BMI: Body Mass Index; PP: Prickly Pear; YYIRT: Yo-Yo Intermitted Recovery Test; GLU: Blood Glucose; NS: Not Significant; NR: Not Reported; CHO: Carbohydrate; h-hours; INS: Serum insulin; ↓: Decrease; ↑: Increase.

**Table 2:** The summary of the effect of *Opuntia spp*. cladode consumption on blood glucose and insulin in human trials [43,44,45,46,47,48,49,50,51,52,53,54,55].

Author (Year)	Participant, Sample Size	Aim	Intervention	Relevant Outcomes	Results (Treatment vs. Placebo)
**Acute Results**					
Frati et al. (1983) [45]	‘Healthy’ Males (*n* = 5) Age: 28–35 years	The effect of ‘Nopal’ (CLD; *Opuntia streptacantha)* consumption on OGTT, on GLU and INS in healthy males	Length: Single consumption Treatment: (1) 75 g GLU (2) 75 g GLU + 100 g broiled CLD stems (20 min before OGTT) Control trial	GLU (mg/dl) INS (µU/mL) Time: 0, 60, 120, 180 min	GLU: ↓ after meal CLD consumption 0 min (*p* < 0.01), 60 min (*p* < 0.05), 120 min (*p* < 0.05), 180 min (*p* < 0.01) INS: ↓ after meal CLD consumption; 0 min (*p* < 0.01), 60 min (*p* < 0.01), 120 min (*p* < 0.01), 180 min (*p* < 0.02) T2DM
Frati et al. (1987) [48]	‘Healthy’ Adults (*n* = 16) Group 1: (*n* = 5) Group 2: (*n* = 6) Group 3: *(n* = 5)	The effect of CLD *(Opuntia sp.)* consumption on blood GLU and INS response, to dextrose in OGTT	Length: Single Consumption Group 1: 12 h fasted + treatment Group 2: OGTT (25 g GLU), CLD given after time 0, before GLU load Group 3: OGTT (25 g GLU) + treatment CLD Treatment: 100 g on CLD, ground and mixed with 100 g of water Controls in each group: 12 h fasting: OGTT + Nopal load OGTT + 200 mL water	GLU INS Blood collection: Group 1 and 3: 0, 30, 60, 120, 180 min Group 2: 0, 5, 15, 30, 60, 120 min	CLD consumption on GLU and INS = NS (*p* > 0.05) GLU: NS (*p* > 0.05); except for: Group 1 vs Group 2 (CLD vs. OGTT+CLD): 60 min; 180 min (*p* < 0.025)
Frati et al. (1990) [54]	T2DM (*n* = 8; 2 Males; 6 Females) Age: 45–68 years (Mean: 55 years)	The effect of *Opuntia ficus indica* on hyperglycemia (CLD) in T2DM	Length: single consumption Treatments: (500 g) (1) Entire Broiled CLD (2) Blended and broiled CLD (3) Blended CLD (4) Blended and heated (60 °C) CLD Cross-over trial	Fasted (12 h) blood: 40, 60, 120, 180 min GLU	↓ GLU at 120 min and 190 min (VS. control (baseline); *p* < 0.01) NS differences between heated and unheated (*p* > 0.05)
Frati et al. (1991) [44]	Group 1: (*n* = 8; 2 Males; 5 Females) T2DM Age:36–65 years (50 years) Group 2: (*n* = 6; 3 Females; 3 Male) ‘Healthy’ volunteers Age: 15–45 years (25 years)	The effect of two sequential doses of *Opuntia streptacantha* stems blood GLU and INS	Length: single consumption Treatment: 500 g broiled cladode Test A: Ingestion at time 0 h, 2 h; 300 mL water at 4 h Test B: Ingestion at time 0 h, 300 mL water at 2 h, 4 h Test C (control): 300mL water at 2 h, 4 h Control-trial	Blood: 0,2,4 and 6 h GLU	Test A vs Test B: ↓GLU: Group 1 first ingestion at 2, 4 and 6 h (*p* < 0.001) NS: ‘Healthy’ participants (*p* > 0.05)
Frati et al. (1991) [43]	Group 1: T2DM (*n =*14; 9 Males, 5 Females) Age: 32–56 years (Mean: 43.4 years); Group 2: ‘Healthy’ (*n* = 14; 9 Males; 5 Females) Age: 23–51 years (Mean: 32.7 years)	The effect of ‘Nopal’ (CLD; *Opuntia streptacantha)* consumption on blood and GLU of T2DM	Length: Single consumption Group 1 and 2 treatments: (fasted) 500g grilled CLD *(Opuntia streptacantha);* or 400 mL Water Randomized control trial	GLU INS	GLU: ↓ in T2DM (Vs Control) (p *<* 0.005, 60 min; *p* < 0.005 120; p<0.001 180 min) ‘Healthy’- NS (*p* > 0.05) INS: (*n* = 7, each Group) ↓- T2DM (vs. control) (*p* < 0.001; 120 min, 180 min) ‘Healthy’: NS
Frati et al. (1988) [47]	T2DM (*n* = 16) Group 1: (*n* = 16; 9 Males, 7 Females) Age: 32–67 years, Mean: 48.3±11.4 years (*n* = 12 received sulfonylureas) Group 2: (*n* = 10; 6 Males; 4 Females) Age: 31–67 years Mean: 46.2 ± 10.8 y; (*n* = 10 received sulfonylureas) Group 3: (*n* = 6; 4 Males; 2 Females) Age: 33–66 years Mean: 48 ± 11.7 years (*n* = 4 received sulfonylureas)	The effect of CLD *(Opuntia streptacantha)* consumption on T2DM	Length: Single consumption Hyperglycemic agents discontinued 72 h before trial. Treatment: Group 1: 500 g of Broiled CLD Group 2: 300 mL water Group 3: 500 g of Broiled Squash (zucchini) Randomized control-trial	GLU INS (fasted; 12 h) Time: 0, 60, 120, 180 min.	GLU: ↓ (vs. basal; *p* < 0.001) *Group 1* vs. *2:* ↓ 60 min (*p*< 0.025); 120 min (*p* < 0.001); 180 min (*p* < 0.001) Group 2: No change Group 3: NS (*p* > 0.05) INS: ↓ Serum INS Group 1 vs. 2: ↓ 120 min, 180 min (*p* < 0.001) Group 3: NS (*p* > 0.05)
Lopez-Romero et al. (2014) [50]	Study 1: ‘Healthy’ (*n* = 7; 3 Males, 4 Females) Age: 26.3 ± 1.2 years; BMI: 23.5 ± 0.8 kg/m^2^Study 2: Group 1: T2DM (*n* = 14; 4 Males; 10 Females) Age: 40–60 years (Mean 48 ± 2.1 years); BMI: <30 kg/m^2^ (Mean 28.9 ± 1 kg/m^2^) Group 2: ‘Healthy’ (*n* = 7; 4 Males; 3 Females) Age: 25–54 years (Mean 21.1 ± 1.2 years) BMI: <25 kg/m^2^ (Mean: 22.2 ± 0.6 kg/m^2^)	Determine glycemic index of Nopal and Effect of nopal consumption on blood GLU	Fasted (12 h) Treatment: 150 g steamed Nopal Study 1: Length: Single consumption Dried nopal (CLD): 24.8% CHO; 32.2% insoluble fiber; 4.8% soluble fiber; <1.9% fat; 15.4% PRO. 50 g of Nopal vs. 50 g GLU Control trial Study 2: Length: Single consumption *High CHO breakfast*: 300 kcal; 89% CHO; 6% PRO; 5% fat (240 mL apple juice, 55.6 g white bread, 21 g strawberry jam) *High Soy Protein Breakfast*: 344 kcal; 42.2% CHO, 40.7% PRO; 16.9% fat (61.5 g soy hamburger, 230 mL soymilk beverage). Washout period: 1 week Cross-over trial	GLU INS Study 1: Blood (capillary and Venous) 15, 30, 45, 60, 90 and 120 min Study 2: Blood (capillary and Venous) 15, 30, 45, 60, 90, 120 and 150 min	Study 1: *Nopal ingestion (vs. GLU)* ↓ AUC GLU (*p* < 0.001) *Glycemic index on Nopal*: 32.5 ± 4.0 *Nopal ingestion (vs. GLU)* ↓ AUC INS (*p* < 0.05) *Insulinemic index of Nopal*: 36.1 ± 6.1 Study 2: *High CHO Breakfast + Nopal*= ↓ GLU at 30 min (*p* < 0.05), 45 min (*p* < 0.01), 60 min (*p* < 0.01); ↓ AUC GLU (*p* < 0.001). ↓ AUC of INS (*p* < 0.05) in diabetes patients *High soy protein breakfast:* ↓GLU (*p* < 0.001) regardless of group; Nopal GLU was NS (*p* > 0.05) Nopal INS was NS (*p* > 0.05)
Castaneda-Andrade et al. (1997) [53]	T2DM (*n* =8; 2 Males; 6 Females) Age: 58.4 years (Range: 48.9–67.8 years)	The effect of *Opuntia streptacantha* on hyperglycemia (CLD) in T2DM	Length: Single consumption Treatment: Group A: 250 mg CLD capsule Group B: placebo capsules Randomized cross-over trial	12 h fasted blood (0, 60, 120, 180 min) GLU INS	↓ GLU (60 + min) both groups; ↓ GLU (basal vs. 180 min) (*p* < 0.001) for both groups
**Long-term results**					
Frati et al. (1983) [46]	11 Males; 18 Females (*n* =29) ‘Healthy’: (*n* = 8) ‘0–8% overweight; Mean 8%’ Obese: (*n* = 14) ‘18–70% overweight’; Mean 35% T2DM: (*n* = 7); 1–5% overweight; received tolbutamide during trial	The effect of ‘Nopal’ (CLD; *(Opuntia streptacantha)* consumption on serum lipids, glycemia and body weight	Length: 10 days Treatment: (1) Regular diet (2) Regular diet + 100 g broiled CLD before meals, 3 × day; 10 days Total per day: 300 g CLD Total: 3000 g/10 days Control trial	GLU (m mol/DI) (Fasting; Pre- VS. Post-treatment)	GLU: ↓ (vs. fasting blood GLU) T2DM (*p* < 0.001), ‘non-T2DM’ (*p* < 0.05)
Guevare-Cruz et al. (2012) [49]	‘Healthy’ (*n* = 67) Age: 20–60 years BMI: 25–39.9 kg/m^2^	The effect of dietary pattern on biochemical markers	2 weeks prior: Reduced energy diet, low-saturated fay and low-cholesterol diet. (15 × eating plans: 50–60% CHO; 15% PRO; 25–35% FAT; <7% saturated fat, <200mg/d cholesterol, 20–30 g/d fiber.) Treatment: Length: 2 months Group 1: Controlled Dietary Pattern Group 2: Placebo *Dietary Pattern:* 100 g nopal (CLD), 4 g chia seeds, 22 g oats, 32g soy bean proteins, 0.02 g sweetener (‘Splenda’), 1 g flavoring *Placebo:* 30 g calcium caseinate, 30 g maltodextrin, 0.02 g sweetener (‘Splenda’), 1 g flavoring. Single-center, randomized, placebo-controlled, double-blind, parallel-art study	Pre/Post intervention OGTT: 75 g GLU (120 min) GLU INS	No change in either treatment group for GLU INS AUG: No difference in OGTT. ↑ INS in OGTT of Dietary Pattern (*p* < 0.05) ↑ Significant diet and time interaction between pre/post diet intervention on INS (*p* < 0.01)
Linares et al. (2007) [52]	Females (*n* = 59) Age: <35 years (10.29%) 35–45 years (27.94%) 45–55 years (41.18%) >55 years (20.59%) BMI: 25–40 kg/m^2^ Placebo group: *n* = 33 Treatment group: *n* = 35		Length: 6 weeks- with ‘balanced diet’ (2000 kcal; 38% Fats; 17% PRO; 45% CHO) and 30 min of physical activity/day. Treatment: 3 × 1.6 g ‘NeOpuntia^©^’ capsules, after meal/day with water. Control: 3 × Placebo Capsule Monocentric, randomized, double-blind, placebo-controlled study	Prevalence of Fasting GLU (>0.95 g/L)	GLU: Treatment group remained the same during study period (*n =* 15). ↑ in placebo group (*n* = 20→22).
Bacardi-Gascon et al. (2007) [55]	T2DM (*n* = 36) Age: 47–72 years GLU: 8.0 ± 2 mmol/L BMI: 24.86 ± 3.76 kg/m^2^ Group 1: *n* = 11 Group 2: *n* = 9 Group 3: *n* = 9	Post-prandial glycemic response to nopal (CLD)	Length: 3 weeks Treatment: 1 × week/3 weeks (fasted) (1) Chilaquiles (corn tortilla, vegetable oil, tomato sauce, cheese, pinto beans) (with vs with-out CLD) (2) Burrito (scrambled egg, tomato, onion, vegetable oil, flour tortilla, pinto beans) (with and without-CLD) (3) Quesadillas (flour tortilla, low fat cheese, avocado, pinto beans) (with and without CLD) 7-day washout periods Control: Chilaquiles, Burrito and Quesadillas without cladode Placebo-controlled Cross-over trial	Fasted Baseline: 0, 50g of white bread at 15, 30, 45, 60, 90 and 120 min GLU	↓ GLU over all; (*p* = 0.029) Group 1: ↓ AUC (with vs. without; *p* = 0.013) ↓ Glycemic index white bread and GLU (*p* = 0.018) Group 2: ↓ AUC (with vs. without CLD; *p* = 0.011) ↓ Glycemic index of white bread and GLU (*p* = 0.025) Group 3: ↓ AUC (with vs. without CLD; *p* = 0.019) ↓ Glycemic index of white bread and GLU (*p* = 0.027; *p* =0.025)

NNIDM: Non-Insulin Dependent Diabetes Mellitus; BMI: Body Mass Index; CLD: Cladode; GLU: Blood Glucose; INS: Serum insulin; NS: Not Significant; h-hours; OGTT: Oral Glucose Tolerance Test; AUC: Area Under the Curve ↓: Decrease; ↑: Increase.

**Table 3:** The summary of the effect of combined or unidentified *Opuntia spp*. fruit and cladode consumption on blood glucose and insulin in human trials [36,51,56,57].

Author (Year)	Participant, Sample Size	Aim	Intervention	Relevant Outcomes	Results (Treatment vs Placebo)
**Acute Results**					
Deldicque et al. (2013) [56]	‘Healthy’ Males (*n* = 11) Age: 21.1 ± 0.9 years Weight: 74.4 ± 4.2 kg	Effect of: (1) ‘OpunDia’ (2) ‘OpunDia’ + Leu (Leu) Supplementation on INS stimulation	Length: Single Consumption Fasted; post-30 min endurance exercise (70% VO_2_); 75 g of GLU with treatment at: 0 min and 60 min. Treatment: (1) Placebo ‘LUVOS Heilerde’ (2) 1000 mg ‘OpunDia^©^’ (3) 3 g Leu (4) 1000 mg ‘OpunDia^©^’ + 3 g Leu *1-week wash-out* Randomized double-blind cross-over study	OGTT (2 h) INS GLU	OGTT: INS higher with ‘OpunDia+Leu’, after 60 min (vs. Placebo) INS: OpunDia+Leu ↑40% at 2 h (vs. placebo) (*p* < 0.05) OpunDia ↑ 2 h (vs. Placebo) (*p* < 0.05) AUC: OpunDia ↑ (*p* = 0.06) AUC: OpunDia+Leu (*p* < 0.05) Leu-no effect GLU: OpunDia ↓GLU by 7% (90 min), 15% (120 min) (vs. placebo; *p* < 0.05) AUC: ↓ OpunDia vs. placebo (*p* < 0.05)
Van Proeyen et al. (2012) [57]	‘Healthy’ Males (*n =* 6) Age: 21.0 ± 1.6 years; Weight: 78.1 ± 6.0 kg;	The effect of ‘OpunDia’ supplementation on blood GLU and INS before and after exercise	Length: Single consumption *Standardized Dinner:* 860 kcal; 73% CHO, 14% fat, 13% PRO); 12 h Fast *Exercise:* 30 min submaximal endurance Treatment: 500 mg, 1000 mg, 1500 mg ‘OpunDia’Placebo ‘LUVOS Heilerde’ *Pre- and post- exercise:* *OGTT* (2 h)-75 g GLU * Post-exercise OGTT, additional bolus at 60 min *2-week washout period* Double-bind Placebo-controlled crossover study	30, 60, 90, 120 min capillary and Venous blood collection GLU INS	Pre-exercise: ↓GLU: 30 min (*p* < 0.10), 60 min (*p* < 0.02). 60 + min = NS (*p* > 0.05). ↓GLU AUC (*p* < 0.03) ↑ INS (*p* < 0.01). ↑ INS: 30 min (*p* < 0.03) INS AUC = NS (*p* > 0.05) Post-exercise: ↓ GLU: 60 min (*p* < 0.04) ↓ GLU AUC (*p* < 0.03) INS = NS (*p* > 0.05)
Godard et al. (2010) [36]	Obese (*n* = 29; Males and Females) BMI: 30–35 kg/m^2^ Age: 20–50 years Acute: *n* = 29 Chronic: Treatment: *n* = 15 Control: *n* = 14	Determine the acute and chronic effects of ‘OpunDia’	Length: Single consumption Treatment: 200 mg ‘OpunDia’ Placebo: 200 mg microcrystalline cellulose Acute trial: 400 mg ‘OpunDia’ 30 min prior to 75 g GLU (OGTT); Chronic trial: Length: 16 weeks 2 × Treatment (200 mg) or Placebo Randomized, Double-blind, placebo-controlled trial	Acute: GLU Blood time: 0, 30, 60, 90, 120 min Chronic: OGTT GLU INS	Acute: ‘OpunDia’ vs Fasted: 60, 90 and 120 min (*p* < 0.05) Chronic: GLU: Pre/Post- ‘OpunDia’: ↓ 60, 90 and 120 min (*p* < 0.05) Pre/Post Placebo: ↓ 60, 90 min (*p* < 0.05) INS: Pre/Post ‘OpunDia’: NS (*p* > 0.05) Pre/Post Placebo: NS (*p* > 0.05)
**Long-term results**					
Guevara-Arauza et al. (2011) [51]	‘Healthy’ (*n* = 28; 12 Males, 16 Females)	To determine bio-functional effects of nopal (CLD) and PP fruit products	Length: 3 weeks Treatment: Supplement diet with 40 g Bars: Control-bar vs ‘Nopal (32%) with PP pulp Jam’ bar (15 g); and 100 g Tortillas vs. Tortillas with Nopal (48%). Dose: Twice a day, three-weeks. Control trial	Fasted (8 h) blood samples;	Tortilla VS. control: ↓ GLU (*p* < 0.05) Tortilla and PP pulp jam bars: ↓ GLU (*p* < 0.05)

PP: Prickly Pear; OGTT: Oral Glucose Tolerance test; CHO: Carbohydrate; PRO: Protein; GLU: Blood Glucose; INS: Serum insulin; Leu: Leucine; AUC: Area Under the Curve; NS: Not Significant; ↓: Decrease; ↑: Increase; Note: ‘OpunDia^©^’ is a capsule supplement containing 75% Cladode and 25% PP fruit extract (Solvent: Water).

**Table 4:** Risk of bias summary for included studies in this systematic review.

	Selection Bias		Performance Bias	Detection Bias	Attrition Bias	Reporting Bias	Other Bias
	Random Sequence Generation	Allocation Concealment	Blinding of Participants and Personnel	Blinding of Outcome Assessment	Incomplete Outcome Data	Selective Reporting	
**Prickly Pear Fruit**							
Khouloud et al. (2016) [39]	High	Unclear	High	Unclear	Low	Low	Unclear
Wolfram et al. (2002) [40]	High	Unclear	High	Unclear	Low	Low	Unclear
Pimienta et al. (2008) [42]	High	Unclear	High	Unclear	Low	Low	Unclear
Wiese et al. (2004) [41]	Low	Low	Low	Unclear	Low	Low	Unclear
**Cladode/leaf**							
Frati et al. (1983) [45]	Unclear	Unclear	High	Unclear	Low	Low	Unclear
Frati et al. (1983) [46]	Unclear	Unclear	High	Unclear	Low	Low	Unclear
Frati et al. (1987) [48]	Unclear	Unclear	High	Unclear	Low	Low	Unclear
Frati et al. (1991) [43]	High	Unclear	High	High	Low	Low	Unclear
Frati et al. (1991) [44]	Unclear	Unclear	High	Unclear	Low	Low	Unclear
Frati et al. (1988) [47]	High	Unclear	High	High	Low	Low	Unclear
Frati et al. (1990) [54]	High	Unclear	High	Unclear	Low	Low	Unclear
Guevare-Cruz et al. (2012) [49]	Low	Low	Low	High	Low	Low	Unclear
Lopez-Romero et al. (2014) [50]	Unclear	Low	High	Unclear	Low	Low	Unclear
Castaneda-Andrade et al. (1997) [53]	Unclear	Unclear	Low	Unclear	Low	Low	Unclear
Linares et al. (2007) [52]	Unclear	Low	Low	Unclear	Low	Low	Unclear
Bacardi-Gascon et al. (2007) [55]	Low	Unclear	High	Unclear	Low	Low	Unclear
**Combination of Fruit and Cladode/Leaf**							
Deldicque et al. (2013) [56]	Low	Unclear	Low	Unclear	Low	Low	Unclear
Van Proeyen et al. (2012) [57]	Low	Unclear	Low	Unclear	Low	Low	Unclear
Godard et al. (2010) [36]	Low	Unclear	Low	Unclear	Low	Low	Unclear
Guevara-Arauza et al. (2011) [51]	Unclear	Unclear	High	Unclear	Low	Low	Unclear

The forms of bias considered included; selection, performance, detection, attribution, reporting and ‘other’ biases, which were scored either ‘low’, ‘high’ or ‘unclear’.
